# The prevalence of and risk factors for restless legs syndrome: A nationwide study

**DOI:** 10.3389/fpsyt.2022.987689

**Published:** 2023-01-03

**Authors:** Saad Mohammed AlShareef

**Affiliations:** Department of Medicine, College of Medicine, Imam Mohammad Ibn Saud Islamic University, Riyadh, Saudi Arabia

**Keywords:** anxiety, depression, Epworth sleepiness scale, epidemiology, restless legs syndrome (RLS)

## Abstract

**Objectives:**

Restless legs syndrome (RLS) is a neglected diagnosis, and most individuals with RLS do not access effective therapies. There has yet to be a nationwide study of the prevalence of and associated risk factors for RLS in Saudi Arabia.

**Materials and methods:**

A population-wide survey was administered to Saudi Arabian adults to assess RLS prevalence and its association with other clinical and demographic variables. RLS was defined according to 2012 IRLSSG Diagnostic Criteria. Persistent RLS was defined as symptoms occurring more than a few nights each week, and RLS causing significant daytime impairment was defined as symptoms causing “severe” excessive daytime sleepiness measured by the Epworth Sleepiness Scale. Associations were evaluated using univariate analyses and binary logistic regression.

**Results:**

10,106 individuals completed the survey. Persistent RLS was reported in 11.9% of participants, which caused significant daytime impairment in 1.2% of participants. In multivariable analysis, younger age (OR 0.96, 95% CI 0.95–0.97; *p* < 0.001), tobacco smoking (OR 1.28, 95% CI 1.07–1.53; *p* = 0.008), anxiety (OR 1.34–1.42; *p* < 0.05), and moderate to severe depressive symptoms (OR 1.52–2.40; *p* < 0.01) were associated with persistent RLS. Younger age (OR 0.96, 95% CI 0.93–0.99; *p* = 0.015), female gender (OR 2.28, 95% CI 1.32–3.94; *p* = 0.003), and moderately severe to severe depressive symptoms (OR 13.59 and 26.7, respectively; *p* < 0.001) were independently associated with RLS causing significant daytime impairment.

**Conclusion:**

RLS is common in adults in Saudi Arabia and is often co-morbid with moderate to severe depressive symptoms. Both RLS and depression represent a silent epidemic in Saudi Arabia requiring active inquiry by all healthcare workers to reduce their burden and impact.

## 1 Introduction

Despite intensive research efforts over the last 20 years, restless leg syndrome (RLS) remains a neglected diagnosis (1). RLS is a sensorimotor disorder that impacts sleep quality in sufferers and it is characterized by four major symptoms: (i) the urge to move legs, often due to an unpleasant sensation, (ii) more in the evening and night, with (iii) the urge usually relieved by movement and (iv) increased by rest (2). RLS usually responds to dopaminergic therapy, suggesting that the disorder is underpinned by alterations in central dopaminergic transmission and pathways (3).

Despite recognition that RLS is common within the general population, reported in anywhere between 4 and 29% of adults in North America and Europe (4), less than 10% of people experiencing RLS symptoms get a formal diagnosis (5). This problem appears to be particularly acute in Saudi Arabia; in one study, no patient attending primary health clinics and subsequently diagnosed with RLS had been diagnosed or treated for RLS in the past (6). Given this lack of awareness about RLS but that a variety of drugs are available to address symptoms, including dopaminergic agents, opioids, anticonvulsants, and sedative hypnotics, it is therefore important to quantify the extent of the problem in different countries to inform local practice. Although there are some data on the estimated prevalence of RLS in Saudi Arabia from local studies on specific populations (6, 7), there has yet to be a nationwide study of the prevalence and associated risk factors for RLS in Saudi Arabia.

To fill this knowledge gap, this study investigated the prevalence of RLS in Saudi Arabia and its association with other clinical and demographic variables by gathering data through a population-wide survey of adults across the country. This first population-wide evaluation of RLS in Saudi Arabia not only highlights that this condition is common but also that it is frequently co-morbid with a silent epidemic of mental health problems. Knowledge of the co-existence and frequency of these conditions could provide the rationale for optimized pharmacotherapy in affected individuals.

## 2 Materials and methods

### 2.1 Population and study survey

This study population and the survey have been described in previous publications (8, 9). Briefly, Saudi Arabian adults aged 18 years and over were contacted by a research and marketing organization between November 6, 2019 and December 6, 2019 and invited by email and telephone alerts to complete the survey online. The sample was randomly drawn from a large panel database of the Saudi population in the Saudi Telecom Company database representing all thirteen Saudi provinces and representing different age groups, gender, and location. The purpose of the research was mentioned in the primary message along with the principal investigator details. Electronic consent was obtained from each participant before participation, and the Institutional Review Board of Imam Mohammad Ibn Saud Islamic University approved the study protocol.

### 2.2 Study questionnaire

The questionnaire was designed based on the study objectives and previously published survey instruments to assess: (i) study population demographics (gender, age, height, weight, marital status); (ii) generalized anxiety disorder using the validated GAD-7 questionnaire with scores of 5, 10, and 15 taken as the cut-off points for mild, moderate, and severe anxiety, respectively (10); (iii) depression severity using the validated Patient Health Questionnaire (PHQ)-9 questionnaire with scores of 5, 10, 15, and 20 taken as the cut-off points for normal, mild, moderate, moderately-severe, and severe depression severity, respectively (11); (iv) Epworth sleepiness scale (ESS) using the validated eight-item questionnaire assessing the likelihood of falling asleep during a variety of daily living situations (subcategorized into 0–10 normal daytime sleepiness, 11–12 mild excessive daytime sleepiness, 13–15 moderate excessive daytime sleepiness, 16–24 severe excessive daytime sleepiness) (12); and (v) RLS defined according to the 2012 Revised International Restless Legs Syndrome Study Group (IRLSSG) Diagnostic Criteria for RLS (2): (1) an urge to move the legs usually but not always accompanied by or felt to be caused by uncomfortable and unpleasant sensations in the legs; (2) beginning or worsening during periods of rest or inactivity such as lying down or sitting; (3) partially or totally relieved by movement, such as walking or stretching, at least as long as the activity continues; (4) only occurring or worse in the evening or night than during the day; and (5) not solely accounted for as symptoms primary to another medical or a behavioral condition (e.g., myalgia, venous stasis, leg edema, arthritis, leg cramps, positional discomfort, habitual foot tapping). Participants were asked about the frequency of their symptoms over the last month and were defined as having persistent RLS if they experienced their symptoms more than a few nights each week (over the last month) and as having RLS causing significant daytime impairment if the symptoms of RLS caused significant impairment in function in the form of “severe” excessive daytime sleepiness measured by the ESS (i.e., a score of 16–24). The questionnaire was administered in Arabic, and the validated Arabic version of the ESS (13), GAD-7 (14), and PHQ-9 were used (14). Persistent RLS and RLS causing significant daytime impairment were the outcomes of interest.

### 2.3 Statistical analysis

Participant demographics were analyzed using descriptive statistics with means [± standard deviation (SD)] for continuous variables and counts (with percentages) for categorical variables. For univariate analysis, differences between continuous variables (age) were assessed using Student’s *t*-test and differences between categorical variables were assessed using the chi-squared test. Parameters with *p*-values < 0.25 in univariate analysis were used to design the most appropriate binary logistic regression model for each outcome variable, since lower *p*-value thresholds such as 0.05 can fail to identify variables known to be important (15). There were no strong intercorrelations between variables. Odds ratios were calculated with 95% confidence intervals (CIs). A *p*-value < 0.05 was considered statistically significant. All analyses were performed using IMS SPSS Statistics v28 (IBM Statistics, Armonk, NJ, USA).

## 3 Results

### 3.1 Overall population and prevalence of restless leg syndrome

A total of 10,106 individuals completed all or part of the survey. The demographics of the employed survey respondents are shown in Table 1. The average age of the study population was 30.7 ± 11.3 years, and 52.7% of respondents were female. Closely reflecting published obesity statistics for Saudi Arabia (16), there was a high prevalence of obesity in the study population (31.7%). The majority of participants were single (53.6%), only 13% were tobacco smokers, and there was a high prevalence of self-reported migraine in the study population (36.3%). The GAD-7 and PHQ-9 mental health screening tools for anxiety and depression detected severe symptoms in 13.9 and 10.7% of the study population. Overall, about a third (34.1%) and nearly a half (46.2%) of respondents reported moderate or greater anxiety and depression, respectively.

**TABLE 1:** Demographics of the survey respondents and the prevalence of restless legs syndrome (RLS).

Variable		Number	Percentage or mean ± SD
Age (years)		8617	30.7 (11.3)
Gender	Male	4089	47.3
	Female	4560	52.7
BMI	Underweight (< 18.5)	1075	12.7
	Healthy (18.5–24.9)	2733	32.2
	Overweight (25–29.9)	1989	23.4
	Obese	2689	31.7
Marital status	Married	3699	42.9
	Divorced	254	2.9
	Single	4616	53.6
	Widowed	48	0.6
Smoker	Yes	1316	13.0
	No	8790	87.0
Comorbidities	Migraine	3106	36.3
	Asthma	590	5.8
	COPD	132	1.3
	Thyroid dysfunction	403	4.7
Persistent RLS		1201	11.9
RLS causing significant daytime impairment		124	1.2
GAD-7	Minimal (0–4)	3081	30.5
	Mild (5–9)	3580	35.4
	Moderate (10–14)	2043	20.2
	Severe (≥ 15)	1402	13.9
PHQ-9	Normal (0–4)	2407	23.8
	Mild (5–9)	3019	29.9
	Moderate (10–14)	2147	21.2
	Moderately severe (15–19)	1450	14.3
	Severe (20–27)	1083	10.7

**FIGURE 1:**
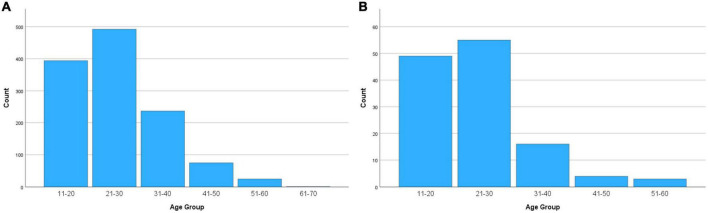
Distribution of **(A)** persistent restless legs syndrome (RLS) and **(B)** RLS causing significant daytime impairment according to age.

Persistent RLS was reported in 11.9% of the study population, and this caused significant daytime impairment in 1.2% of the overall study population. Both persistent RLS and that causing daytime impairment were more common in females (15.8 and 2.2%, respectively) than in males (11.9 and 0.5%, respectively) (Tables 2, 3). The distribution of persistent RLS and that causing daytime impairment according to age group (Figure 1) was also similar, being left skewed and most prevalent in the 21–30 year age group and relatively less common after 31 years of age.

**TABLE 2:** Univariate and multivariate analysis of risk factors for persistent restless legs syndrome (RLS).

Variable		Persistent RLS			
		No [mean ± SD/number (%)]	Yes [mean ± SD/number (%)]	Univariable analysis (*P*-value)	Multivariable analysis (OR (95% CI); *P*-value)
Age (years)		31.4 ± 11.6	26.6 ± 8.8	<0.001	0.96 (0.95–0.97); < 0.001
Gender	Male	3591 (88.1)	483 (11.9)	<0.001	1.14 (0.98–1.34); 0.10
	Female	3829 (84.2)	718 (15.8)		
BMI	Underweight (< 18.5)	873 (81.3)	201 (18.7)	<0.001	Reference
	Healthy (18.5–24.9)	2343 (86.0)	382 (14.0)		0.94 (0.77–1.14); 0.50
	Overweight (25–29.9)	1720 (86.8)	261 (13.2)		1.15 (0.92–1.44); 0.22
	Obese	2348 (87.5)	335 (12.5)		1.18 (0.94–1.48); 0.15
Marital status	Married	3334 (90.4)	353 (9.6)	<0.001	Reference
	Divorced	222 (87.7)	31 (12.3)		1.04 (0.70–1.56); 0.84
	Single	3790 (82.3)	816 (17.7)		0.99 (0.82–1.21); 0.99
	Widowed	47 (97.9)	1 (2.1)		0.33 (0.04–2.40); 0.27
Smoker	No	6472 (86.7)	990 (13.3)	0.007	Reference
	Yes	1101 (83.9)	211 (16.1)		1.28 (1.07–1.53); 0.008
Comorbidities	Migraine	4756 (87.5)	682 (12.5)	<0.001	Reference
		2582 (83.4)	515 (16.6)		0.99 (0.86–1.13); 0.85
	Asthma	6828 (86.0)	1107 (14.0)	0.39	-
		500 (84.7)	90 (15.3)		-
	COPD	7203 (85.9)	1180 (14.1)	1.00	-
		114 (86.4)	18 (13.6)		-
	Thyroid dysfunction	6978 (85.8)	1156 (14.2)	0.11	Reference
		356 (88.8)	45 (11.2)		0.93 (0.67–1.30); 0.68
GAD-7	Minimal (0–4)	2164 (92.4)	178 (7.6)	<0.001	Reference
	Mild (5–9)	2828 (86.9)	428 (13.1)		1.42 (1.16–1.74); < 0.001
	Moderate (10–14)	1567 (83.2)	316 (16.8)		1.34 (1.05–1.70); 0.019
	Severe (≥ 15)	1013 (78.4)	279 (21.6)		1.40 (1.06–1.84); 0.017
PHQ-9	Normal	1567 (93.1)	116 (6.9)	<0.001	Reference
	Mild	2507 (90.3)	268 (9.7)		1.05 (0.82–1.34; 0.71)
	Moderate	1658 (84.5)	304 (15.5)		1.52 (1.17–1.98); 0.002
	Moderately severe	1084 (81.0)	255 (19.0)		1.86 (1.39–2.48); < 0.001
	Severe	757 (74.6)	258 (25.4)		2.40 (1.75–3.29); < 0.001

**TABLE 3:** Univariate and multivariate analysis of risk factors for restless legs syndrome (RLS) causing significant daytime impairment.

Variable		RLS causing significant daytime impairment			
		No [mean ± SD/number (%)]	Yes [mean ± SD/number (%)]	Univariable analysis (*P*-value)	Multivariable analysis (OR (95% CI); *P*-value)
Age (years)		30.8 ± 11.4	24.5 ± 7.8	<0.001	0.96 (0.93–0.99); 0.015
Gender	Male	4064 (99.5)	22 (0.5)	<0.001	Reference
	Female	4457 (97.8)	102 (2.2)		2.28 (1.32–3.94); 0.003
BMI	Underweight (< 18.5)	1046 (97.3)	29 (2.7)	<0.001	Reference
	Healthy (18.5–24.9)	2688 (98.4)	45 (1.6)		0.94 (0.58–1.53); 0.80
	Overweight (25–29.9)	1966 (98.9)	21 (1.1)		0.96 (0.52–1.75); 0.89
	Obese	2663 (99.1)	25 (0.9)		1.12 (0.61–2.08); 0.71
Marital status	Married	3667 (99.2)	29 (0.8)	<0.001	Reference
	Divorced	250 (98.4)	4 (1.6)		1.02 (0.35–2.99); 0.98
	Single	4525 (98.0)	91 (2.0)		0.69 (0.38–1.24); 0.21
	Widowed	48 (100.0)	0 (0.0)		NA
Smoker	Yes	8638 (98.7)	113 (1.3)	0.185	Reference
	No	1305 (99.2)	124 (1.2)		0.74 (0.38–1.44); 0.38
Comorbidities	Migraine	5389 (99.0)	57 (1.0)	<0.001	Reference
		3039 (97.8)	67 (2.2)		1.03 (0.70–1.51); 0.90
	Asthma	7831 (98.5)	120 (1.5)	0.11	Reference
		586 (99.3)	4 (0.7)		0.34 (0.12–1.04); 0.14
	COPD	8277 (98.5)	122 (1.5)	0.72	-
		130 (98.5)	2 (1.5)		-
	Thyroid dysfunction	8032 (98.6)	115 (1.4)	0.19	Reference
		394 (97.8)	9 (2.2)		1.67 (0.81–3.41); 0.16
GAD-7	Minimal (0–4)	3066 (99.6)	11 (0.4)	<0.001	Reference
	Mild (5–9)	3542 (99.2)	27 (0.9)		0.86 (0.40–1.87); 0.70
	Moderate (10–14)	1996 (98.3)	34 (1.7)		0.86 (0.38–1.96); 0.72
	Severe (≥ 15)	1339 (98.8)	52 (3.7)		1.08 (0.46–2.53); 0.86
PHQ-9	Normal	2403 (99.9)	2 (0.1)	<0.001	Reference
	Mild	2997 (99.6)	13 (0.4)		3.32 (0.73–15.19); 0.12
	Moderate	2114 (98.9)	23 (1.1)		6.78 (1.46–31.38); 0.014
	Moderately severe	1410 (31)	23 (2.2)		13.59 (2.89–64.07); < 0.001
	Severe	1019 (94.8)	124 (1.2)		26.7 (5.56–128.13); < 0.001

### 3.2 Associations between demographic and clinical parameters and RLS

In univariable analysis, younger age, being female, lower BMI, being single, migraine, and anxiety and depression were all significantly associated with persistent RLS (Table 2) and RLS causing significant daytime impairment (Table 3) (all *p* < 0.001); tobacco smoking was only associated with persistent RLS (*p* = 0.007). In multivariable analysis, younger age (OR 0.96, 95% CI 0.95–0.97; *p* < 0.001), tobacco smoking (OR 1.28, 95% CI 1.07–1.53; *p* = 0.008), anxiety of any severity (OR 1.34–1.42; *p* < 0.05), and moderate to severe depressive symptoms (OR 1.52–2.40; *p* < 0.01) were independently associated with persistent RLS (Table 2). Younger age (OR 0.96, 95% CI 0.93–0.99; *p* = 0.015), female gender (OR 2.28, 95% CI 1.32–3.94; *p* = 0.003), and moderately severe to severe depressive symptoms (OR 13.59 and 26.7, respectively; *p* < 0.001) were independently associated with RLS causing significant daytime impairment (Table 3).

## 4 Discussion

This study provides new data on the prevalence of RLS in a large representative sample of the adult Saudi Arabian population. Persistent RLS, defined as patients experiencing the essential diagnostic criteria for RLS a few nights a week over the preceding month, was present in 11.9% of the population, consistent with reported general prevalence rates in meta-analyses [between 4 and 29% of adults in North America and Europe (4)] and systematic reviews [between 5 and 14.3% of adults using IRLSSG criteria (17)]. There have only been a few previous studies of RLS in the general Saudi population, with Wali et al. (7) reporting an RLS prevalence of 8.4% in 2,682 adults aged 30–60 and BaHammam et al. (6) reporting an RLS prevalence of 5.2% in 1,303 patients attending primary health care facilities. Other studies in Saudi Arabia have focused on specific groups such as pregnant women (18, 19) or those with end-stage renal disease (20). While we do not know whether or what proportion of these patients meeting diagnostic criteria for RLS were known to the healthcare system for RLS, these data suggest that RLS affects a significant minority of the population and, while perhaps encouraging that only 1.2% of the population had RLS severely affecting daytime sleepiness, RLS is likely to be incurring a serious quality of life cost on individuals and society. Furthermore, our analysis reveals a silent epidemic of mental health problems in the Saudi population, with a about a third and nearly a half of respondents reporting moderate or greater anxiety and depression symptoms, respectively. RLS, anxiety, and depression are a serious but silent threat in Saudi society.

In multivariable analysis, younger age, tobacco smoking, anxiety of any severity, and moderate to severe depressive symptoms were independently and significantly associated with persistent RLS, while younger age, female gender, and moderately severe to severe depressive symptoms were independently and significantly associated with RLS causing significant daytime impairment. In both regression models, depressive symptoms were associated with the greatest risk of RLS, with odds ratios of 1.5–2.4 for persistent RLS and 6.8–26.7 for RLS causing significant daytime impairment, in a dose-dependent manner. The relationship between RLS, mental health disorders, and their functional impact is complex, since patients with RLS often have sleep disturbances and sleep disorder is itself associated with mental health and psychiatric complications (21). Although not every observational study of risk factors for RLS have examined its association with mental health disorders, several studies have reported that patients with RLS have significantly greater anxiety (22) and/or depression symptoms (22, 23), and, conversely, RLS rates as high as 27% have been reported in patients with unipolar depression presenting to a psychiatric clinic (24). Although some cross-sectional studies and case reports suggest that antidepressant use is associated with the onset or worsening of RLS, a systematic review found that, overall, the worsening of RLS symptoms is unlikely following the prescription of an antidepressant, although some antidepressants may be more prone to symptom modification (25). Regardless of cause or effect, RLS and depression/anxiety are often comorbid, and given the probable bi-directional interaction between the two conditions and their potentially serious impact on the patient, enquiring about both disorders is important so that both can be recognized and actively managed as part of holistic care. Furthermore, since dopamine is implicated in the pathophysiology of depression and treatment of RLS may improve depressive symptoms in affected patients (3), the overlap between the two disorders may be exploitable when considering the optimal pharmacological therapy for co-morbid individuals.

While the effect sizes of the other significant variables were generally less than those for anxiety and depression in particular, gender, age, and tobacco use have all been linked to RLS in various studies. Most other studies report that, while RLS can start at any age, most individuals with RLS are over 40 and the risk of RLS increases with age (17). Many studies have also reported that RLS prevalence increase with age, although others have reported a peak prevalence between 30 and 40 years (26). Furthermore, the association with older age appears to be stronger in North American and European populations than in Asian countries (17). Our finding that both persistent RLS and RLS causing significant daytime impairment were associated with younger individuals is unusual; however, overall, our study population was young (mean 30 years), so this result may reflect the distribution of the condition in this demographic. Alternatively, it may reflect the unique features of the condition in this particular Middle Eastern ethnic group, and since there is a known genetic predisposition to RLS and it can be familial (27), it is not unreasonable to hypothesize that there may be geographic, ethnic, or cultural differences in the clinical features of the disease (28); indeed, familial RLS has an earlier age of onset (27) and modifiable lifestyle factors such as caffeine consumption and diet are known to play important roles in RLS risk (29). Regardless, RLS is common even in younger individuals in Saudi Arabia and should therefore not be viewed as solely a disease of the elderly. Most published studies have reported a higher prevalence of RLS in women than in men (17), although interestingly other studies from Saudi Arabia have either reported a higher prevalence in men (7) or an equal male:female distribution (6). This might explain why a gender difference was only seen for those with RLS causing significant daytime impairment in our cohort. Smoking has similarly been reported as an independent risk factor for RLS in several studies (29, 30), although the neurobiological basis for this association remains uncertain. As noted above, RLS is sometimes alleviated with dopaminergic drugs (3), and D2 receptors are postulated to be involved in its pathogenesis. Since nicotine and dopamine interact (31), nicotine may have a direct effect on the neurobiology of the disorder and directly contribute to risk; however, it should be noted that there are reported examples of RLS being alleviated with smoking (32, 33), and the exact nature of this interaction still requires clarification.

Given that this was an observational, cross-sectional study, it is impossible to say whether our finding that different variables were associated with persistent RLS and RLS causing significant daytime impairment was due to a genuine difference in predisposing risk factors for different types and severities of RLS or whether severe, impairing RLS affects different groups in different ways. Indeed, all observational cohort studies of this type are limited by not being able to distinguish cause from effect. Nevertheless, the consistency in a close association between mental health disorders and both persistent RLS and RLS causing significant daytime impairment mandate that mental health problems are actively considered when clinicians are faced with RLS patients.

The study has several limitations. This was a self-reporting survey, so there is a possibility of recall and response bias. Cultural factors impact reporting of somatic and psychological symptoms (34), and given the self-reporting nature of the questionnaire, this might have introduced a cultural bias. The available survey sample was from those enrolled in the online panel, and this may have introduced selection bias. In addition, since the purpose of the research was disclosed to prospective participants, this might have resulted in responder bias. As noted above, this cohort represented a young population with a slight overrepresentation of females, so this might have introduced bias: secondary RLS has been associated with iron (35) and vitamin D deficiency (36), which are more common in females, although the association between vitamin D deficiency and RLS is not well-established [even contradictory (37)] and requires further investigation. Furthermore, there is evidence to suggest that pregnancy can significantly bias RLS prevalence statistics, especially in familial forms (38); future studies should enquire about pregnancy status. Nevertheless, we have confidence that the survey is representative of the wider population, since the young median age of the sample (29 vs. 29.9 years) and high obesity prevalence (32 vs. 35.4%) are highly consistent with key sociodemographic statistics for Saudi Arabia (39). As noted above and for all cross-sectional studies, causality cannot be inferred.

While we did apply differential diagnostic criteria when diagnosing RLS as per IRLSSG Diagnostic Criteria (2), we only asked about symptom frequency in the preceding month rather than over the preceding year, so we were unable to determine the chronicity of RLS in our cohort. Also, we did not ask exactly how many nights each week patients experienced symptoms (the questionnaire asked about symptoms “a few nights a week” or “almost every night”), so our category of persistent RLS was a close approximation to the IRLSSG Diagnostic Criteria category of chronic-persistent RLS, defined as symptoms occurring at least twice weekly. Furthermore, it is possible that there was some misclassification of RLS cases given that the diagnosis was inferred from self-reported data rather than diagnoses made by professionals in the clinical context, which would ensure that other important differential diagnoses were excluded. Finally, the survey did not capture all covariates that might be comorbid with RLS, such as other sleep disturbances or the presence of obstructive sleep apnea, nor was every possible risk factor (such as pregnancy, renal failure, hematological disturbances, or cardiovascular comorbidities) captured in the data; nevertheless, these data provide further evidence of the public health burden of RLS, and its risk factors, and other mental health problems in Saudi Arabia.

Nevertheless, this is the first population-wide study of RLS in Saudi Arabia and is one of the largest cross-sectional population-based studies of its kind. It therefore provides a comprehensive portrait of the prevalence of RLS in Saudi Arabia and some of the risk factors for it. RLS is common in adults in Saudi Arabia, even in the younger age groups, and it is often co-morbid with moderate to severe depressive symptoms, particularly in individuals with RLS causing significant daytime impairment. Both RLS and depression represent a silent epidemic in Saudi Arabia and, given the negative health and social consequences of these conditions but the availability of therapies to manage both conditions, all healthcare workers have a duty to enquire about both RLS and mental health symptoms in every patient.

## Data availability statement

The raw data supporting the conclusions of this article will be made available by the authors, without undue reservation.

## Ethics statement

The studies involving human participants were reviewed and approved by Imam Mohammad Ibn Saud Islamic University (IMSIU), Riyadh, Saudi Arabia. The patients/participants provided their written informed consent to participate in this study.

## Author contributions

The author confirms being the sole contributor of this work and has approved it for publication.
